# Light curve analysis and evolutionary status of four newly identified short-period eclipsing binaries

**DOI:** 10.1038/s41598-024-54289-1

**Published:** 2024-02-18

**Authors:** Mohamed S. Darwish, Ali G. A. Abdelkawy, Gamal M. Hamed

**Affiliations:** 1 Astronomy Department, National Research Institute of Astronomy and Geophysics (NRIAG), Helwan, 11421 Cairo, Egypt; 2 Department of Astronomy and Meteorology, Faculty of Science Al-Azhar University, 11884 Cairo, Egypt

**Keywords:** Mass-luminosity relation, Evolutionary status, Eclipsing binaries, W UMa, Astronomy and astrophysics, Space physics

## Abstract

We present the physical and orbital parameters of four short-period eclipsing W UMa systems: $ZTFJ000030.44 +391106.9$ (referred to as S1), $ZTFJ000817.08+402532.1$ (referred to as S2), $ZTFJ002158.44+252934.04$ (referred to as S3), and $ZTFJ003357.62+415747.8$ (referred to as S4). The absolute parameters and evolutionary status of these systems are determined, and new times of minima are calculated. Additionally, we present the 3D fill-out configuration for each system. The four Systems exhibit moderate contact W UMa binary with a fill-out factor of 49%, 38%, 28%, and 51%, respectively. Comparing the systems’ periods, we observed a proportional relationship, where shorter periods correspond to lower fill-out factors, and longer periods were associated with higher fill-out factors. Based on the derived surface temperatures and mass ratios of the components, all systems are classified as A-type W UMa binaries. The obtained parameters in addition to a list of previously published data are then utilized to derive an updated Mass-Luminosity relation (M-L) for both A and W-type eclipsing W UMa systems. A comparison with previously published relations reveals that the majority of the EW systems lie between 0.2 and 2 M_sun_ on the M-L diagram. Moreover, we discuss the dynamical evolutionary aspects and evolutionary status of the four components, along with their positions on the Zero Age Main Sequence (ZAMS) and Terminal Age Main Sequence (TAMS).

## Introduction

Eclipsing binaries, particularly W UMa-type systems, are widely recognized as valuable tools for understanding stellar evolution and the characteristics of their associated regions. They are characterized by their short period (< 1 day), late spectral type (F, G and K) with low metallicity (e.g.,^1,2^). The light curve of W UMa systems typically displays nearly equal minima, a characteristic that can be explained by the presence of two components with comparable effective temperatures (e.g.^3,4^). The study of such objects allows us to explore various physical and dynamic phenomena, including mass transfer, mass loss, surface magnetic activity of the components, and properties of angular momentum loss. Despite the detection of numerous W UMa systems by surveys like the SuperWASP (Wide Angle Search for Planets)^5,6^, the Zwicky Transient Facility (ZTF^7^) and GAIA^8^, the number of systems that have been thoroughly studied remains relatively small.

In this context, numerous studies have focused on short-period binary systems ($P < 0.3$ days) (e.g.,^9–18^). Although low-mass stars are abundant, further investigation is needed to fully understand how they evolve in close binary systems. Several authors, including^19^, have reported a period cut-off for these systems, typically around 0.22 days. Rucinski^20^ initially suggested that this cut-off was due to stars reaching their fully convective state. However, others like^21^ proposed that the cut-off results from magnetic wind-driven angular momentum loss mechanisms and is connected to the finite age of the binary population. The exact processes behind this phenomenon remain poorly understood, emphasizing the importance of studying systems with $P < 0.3$ days. The present work aims to provide the first comprehensive light curve analysis, encompassing physical, geometrical, absolute parameters, and the evolutionary status of four newly identified ZTF contact binaries (ZTFJ000030.44+391106.9, ZTFJ000817.08+402532.1, ZTFJ002158.44+252934.0, and ZTFJ003357.62+415747.8). Additionally, the study investigates the Mass-Luminosity (M-L) relation for EW systems, including our analyzed systems^22–26^. Data acquisition and the determination of new times of minima for the systems are presented in “Data” section. The results and discussion are provided in “Results and discussion” section, and finally, the conclusion is drawn in “Conclusion” section.

**Table 1:** Names, coordinates and color index (J-H) of the four studied systems.

Star ID	Star name	RA (J2000.0) (deg)	Dec (J2000.0) (deg)	$J-H_{\text {mag}}$
S1	ZTFJ000030.44+391106.9	0.12684	39.18525	0.399
S2	ZTFJ000817.08+402532.1	2.07119	40.42559	0.226
S3	ZTFJ002158.44+252934.0	5.49351	25.49279	0.487
S4	ZTFJ003357.62+415747.8	8.49012	41.9633	0.322

## Data

The photometric observations in this study were obtained from the ZTF database, accessible at https://www.ztf.caltech.edu/ztf-public-releases.html, using the *gri* filters. The collected light curves are generated by using measurements from calibrated single-exposure PSF-fit-derived catalogs. This approach guarantees that there is no contamination of light from nearby systems. Details about the ZTF data reduction can be found at^27^. We conducted a search in the ZTF catalog for variable stars as introduced by^28^. Subsequently, we visually inspected individual light curves of eclipsing binaries and gathered photometric data for systems meeting specific criteria, including: (1) Systems that were conclusively identified as eclipsing binary systems by^27^. (2) Systems that had not been previously analyzed. (3) Systems with orbital periods shorter than 0.3 days, selected because they could be potential contact binaries. (4) Systems for which the visual magnitude and coordinates were suitable for follow-up observations using the Kottamia Astronomical Telescope (a 1.88 m telescope operated by NRIAG). (5) Systems with light curves exhibiting well-defined photometric variations. After collecting data from the ZTF catalogue, it underwent careful curation, which involved visual inspection to identify and subsequently remove outlier data points. Specific details about the selected objects are available in Table 1. The period of the four systems is determined by^28^. To test and refine the period estimation, we applied the Lomb-Scargle Periodogram method^29,30^ on the collected data.

**Figure 1:**
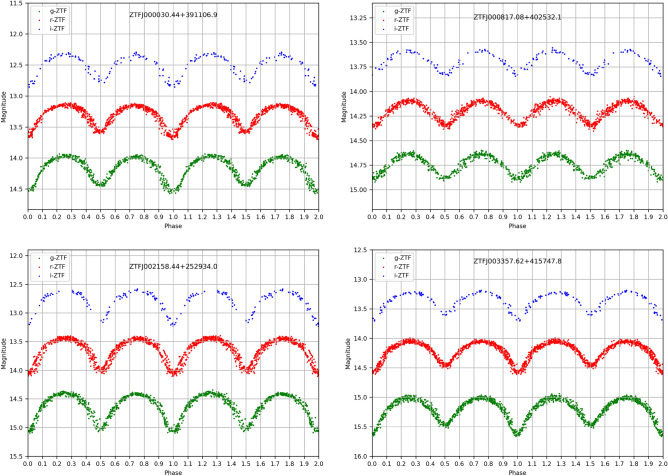
Phase-magnitude plots of the four systems at *gri* filters.

**Table 2:** Times of primary and secondary minima of the four systems.

Star ID	Minima I	Minima II
S1	2,458,471.102500	2,458,366.407095
	2,458,786.16272	2,458,743.393449
	2459,055.430567	2,459,041.462164
	2,459,430.415857	2,459,428.449572
S2	2,458,325.451157	2,458,437.224931
	2,458,694.447419	2,458,714.470023
	2,458,873.118182	2,458,895.134224
	2,459,411.412407	2,459,524.326944
S3	2,458,382.352454	2,458,334.442674
	2,458,699.392269	2,458,812.243322
	2,458,993.464120	2,459,061.438982
	2,459,521.159375	2,459,488.298484
S4	2,458,364.340741	2,458,367.387361
	2,458,771.131667	2,458,737.328287
	2,459,180.243565	2,458,871.090648
	2,459,427.455000	2,459,345.482963

For each of the four systems, we determined the primary and secondary minima timings using the method described by^31^. Our calculations yielded four primary and four secondary minima for each system, as shown in Table 2. We then computed their average values to establish the ephemeris formula. The average primary and secondary minima are provided in Table 3. Table 3 also presents the previously estimated periods alongside those estimated in this study, with discrepancies typically occurring in the fifth decimal place. The phase magnitude diagram for each system is shown in Fig. 1. These values hold significance for future research, enabling the calculation of period changes and the construction of O-C diagrams. Subsequently, we employed the times of primary minima (epoch) ($HJD\_{Min}$) to formulate the ephemeris equations (1, 2, 3, and 4) for each system.

**Table 3:** The average Time of minima, cataloged period, and period from the current work.

Star ID	Min I	Min II	Period	Period (this study)
S1	2,458,712.429444(± 0.002)	2,459,271.856560(± 0.001)	0.287994	0.287993 (± 0.00001)
S2	2,458,426.2912269 (± 0.001)	2,458,651.692909 (± 0.003)	0.284062	0.284060 (± 0.000012)
S3	2,459,323.701782(± 0.003)	2,459,061.438982 (± 0.0015)	0.263978	0.263980 (± 0.000014)
S4	2,458,715.421877(± 0.001)	2,458,541.766962 (± 0.002)	0.290149	0.290150 (± 0.000012)

1 $$\begin{aligned} HJD_{Min} & = 2458712.429444 (\pm 0.002)+ 0.287993^{d} \times E \end{aligned}$$

2 $$\begin{aligned} HJD_{Min} & = 2458426.2912269 (\pm 0.001)+ 0.28406^{d} \times E \end{aligned}$$

3 $$\begin{aligned} HJD_{Min} &= 2459323.701782 (\pm 0.003)+ 0.26398^{d} \times E \end{aligned}$$

4 $$\begin{aligned} HJD_{Min} & = 2458715.421877 (\pm 0.001)+ 0.29015^{d} \times E \end{aligned}$$

The symbol “E” in the equations denotes the number of integer cycles.

## Results and discussion

### Light curve analysis

The light curve analysis of the four systems was performed using PHOEBE code^32^. To determine the surface temperature of the primary component ($T_1$) for each system, we followed a two-step process. Firstly, we estimated the color index J-H (e.g., from the 2MASS catalogue) and determined its corresponding temperature using the color-temperature calibration method outlined in^33^. Secondly, we integrated temperature data from Gaia DR3. Finally, we computed the mean value of these temperature estimates. This temperature was fixed through the light curve modeling. Gravity darkening and bolometric albedo exponents for the effective temperature below 7500K (i.e. convective envelopes) were fixed at $g_1 = g_2 = 0.32$^34^ and $A_1 = A_2 = 0.5$^35^, respectively.

**Figure 2:**
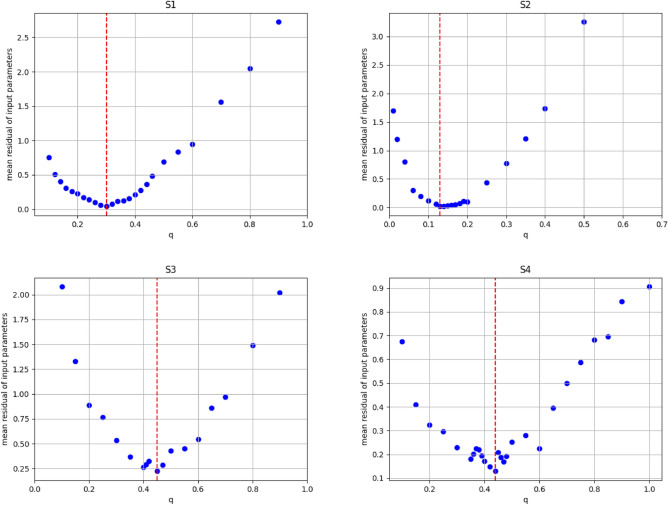
Relation between the mean residual of input parameters and the mass ratio q of the four systems. The dashed red line refers to the selected initial mass ratio.

**Figure 3:**
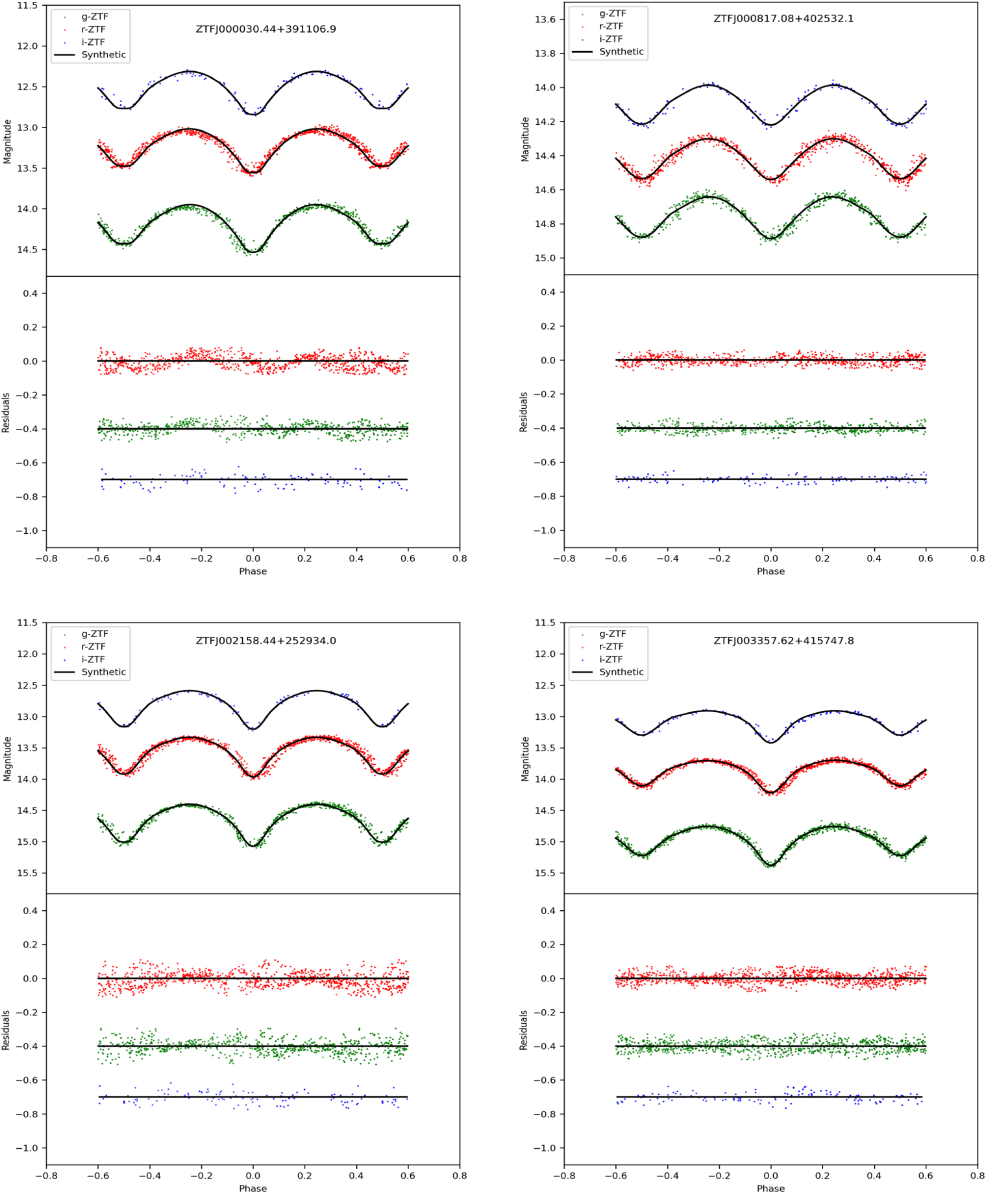
Best-fitted models for the four systems (S1, S2, S3, and S4) are shown. The solid lines represent the synthetic data extracted from the PHOEBE code, while the colored dots depict the observations. The lower panel displays the residuals for each system. The X and Y axes represent the phase and magnitude, respectively.

Logarithmic limb darkening coefficients (X and Y) were derived from tabulated values using the method of^36^. Due to the lack of spectroscopic observations, q-search method (see,^37^) was used to estimate the initial value of the mass ratio ($q = M_2/M_1$) for each system and then adjusted during the light curve fitting. Figure 2 shows the relation between the residual of input parameters and the mass ratio q (for mass-ratio q ranging from 0.1 to 0.9), where a minimal residual occurs at q= 0.31, 0.13, 0.40 and 0.43 for the systems S1, S2, S3 and S4, respectively which were adopted as the initial mass ratio. In this q scan, the primary star’s effective temperature ($T_1$) and the mass ratio q remained fixed, while the secondary star’s effective temperature ($T_2$), orbital inclination *i*, the modified potential ($\Omega _1 = \Omega _2$), and the passband luminosity of the primary ($L_1$) were adjusted. According to the light curve shape (EW), we selected the “Overcontact not in thermal contact” mode in PHOEBE. The adjustable parameters during the fit are $T_2$, $\Omega$, *i*, q and $L_1$. The best fit from the PHOEBE code for each system is shown in Fig. (3). The error associated with these parameters is obtained from PHOEBE code at the best fit model.

Table 4 lists the integrated (*gri*) parameters extracted from the best fit of the light curve modeling for each system.

The W UMa systems in general are classified into two types based on the temperature and mass of their components. In A-type systems, the hotter and brighter star is more massive than its companion, while in W-type systems, the hotter companion is less massive^38–40^. According to the resulting parameters and Following^38^, all the systems were categorized as A-type EW stars. The estimated mass ratio of our sample validates the findings presented by^41^ and^42^, indicating that A-type EW stars generally exhibit a lower mass ratio ($q < 0.72$) than W-type systems. O’Connell effect (asymmetry in the two maxima of the light curve)^43^ was detected only in S4 with a hot spot on the surface of the secondary component. The presence of hot spot region on the surface of the secondary component could be explained by the rapid mass transfer from the primary (e.g.^44–47^). The surface temperature of the spot is found to be 20% higher than the surrounding photosphere.

**Figure 4:**
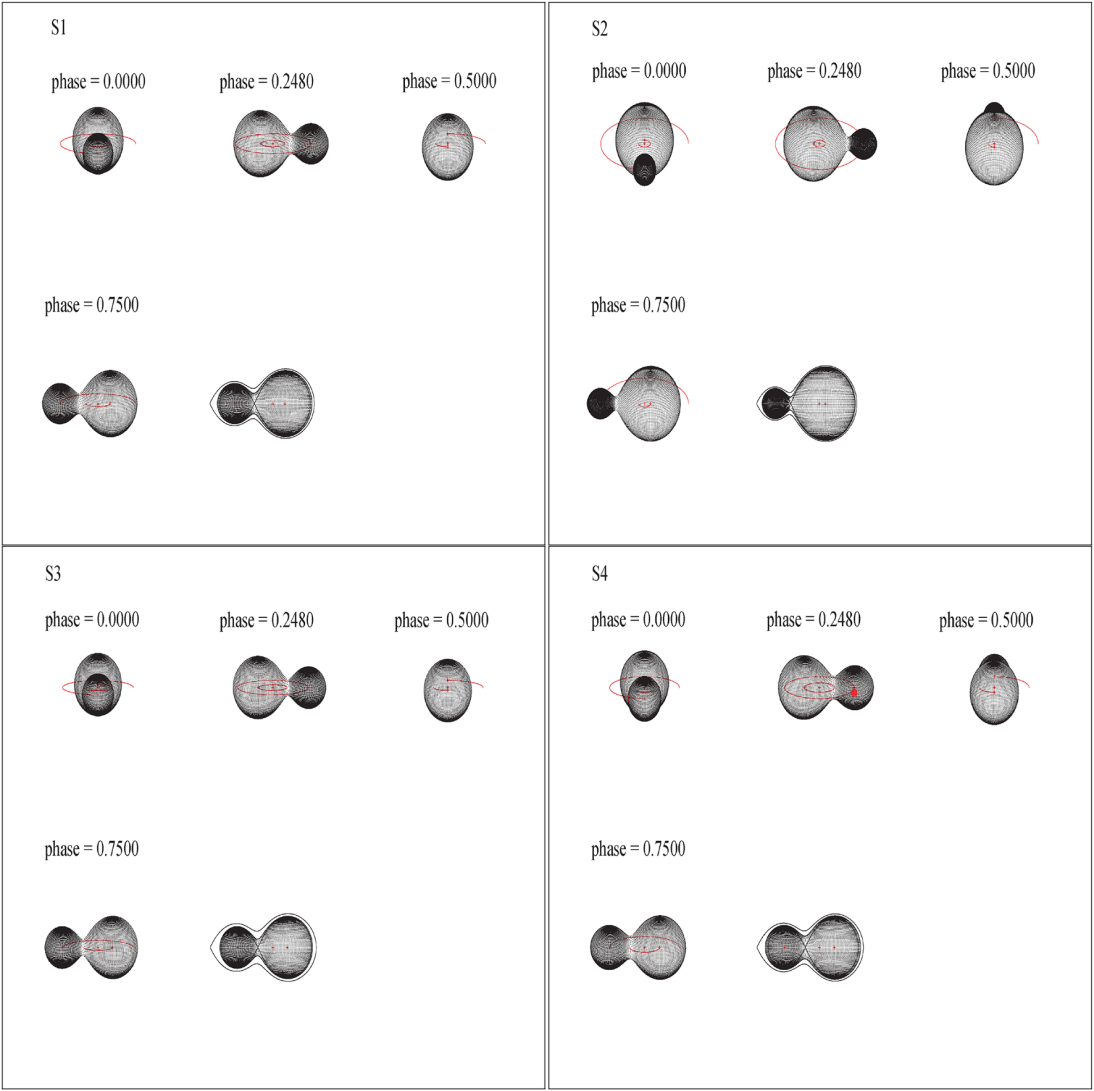
Geometric structure of the systems at different phases. The plots from the left to the right represent stars S1, S2, S3 and S4, respectively. The red dot represents the hot spot on the surface of the second component of star No.4.

**Table 4:** Physical parameters of the four systems extracted from the light curve analysis.

Parameter	S1	S2	S3	S4
$T_1$ (K)	5568.0	6388.0	5081.0	5452.0
$T_2$ (K)	5431 ± 20	6260.0 ± 41	5046.0 ± 16	4912.0 ± 15
*q*	0.3196 ± 0.001	0.1330 ± 0.001	0.4072 ± 0.006	0.4301 ± 0.003
$\Omega _1 = \Omega _2$	2.411 ± 0.005	2.023 ± 0.003	2.622 ± 0.006	2.605 ± 0.005
$g_1 = g_2$	0.32	0.32	0.32	0.32
$A_1 = A_2$	0.5	0.5	0.5	0.5
$X_1 = X_2$	0.776	0.751	0.801	0.801
$Y_1 = Y_2$	0.245	0.252	0.263	0.263
*i* (degrees)	79.34 ± 0.4	65.50 ± 0.2	81.81 ± 0.9	77.06 ± 0.2
$r_{\text {pole1}}$	0.471	0.525	0.444	0.452
$r_{\text {side1}}$	0.512	0.583	0.478	0.488
$r_{\text {back1}}$	0.545	0.605	0.510	0.527
$r_{\text {pole2}}$	0.288	0.217	0.298	0.315
$r_{\text {side2}}$	0.303	0.227	0.313	0.333
$r_{\text {back2}}$	0.359	0.272	0.356	0.391
$L_1 / (L_1 + L_2)$	0.81	0.86	0.69	0.77
Spots parameters	–	–	–	S4
Lat. (deg)	–	–	–	90
Long. (deg)	–	–	–	90
Radius (deg)	–	–	–	10
Temp. fact	–	–	–	1.2
$\sum (o-c)^2$	0.08	0.06	0.11	0.08

**Figure 5:**
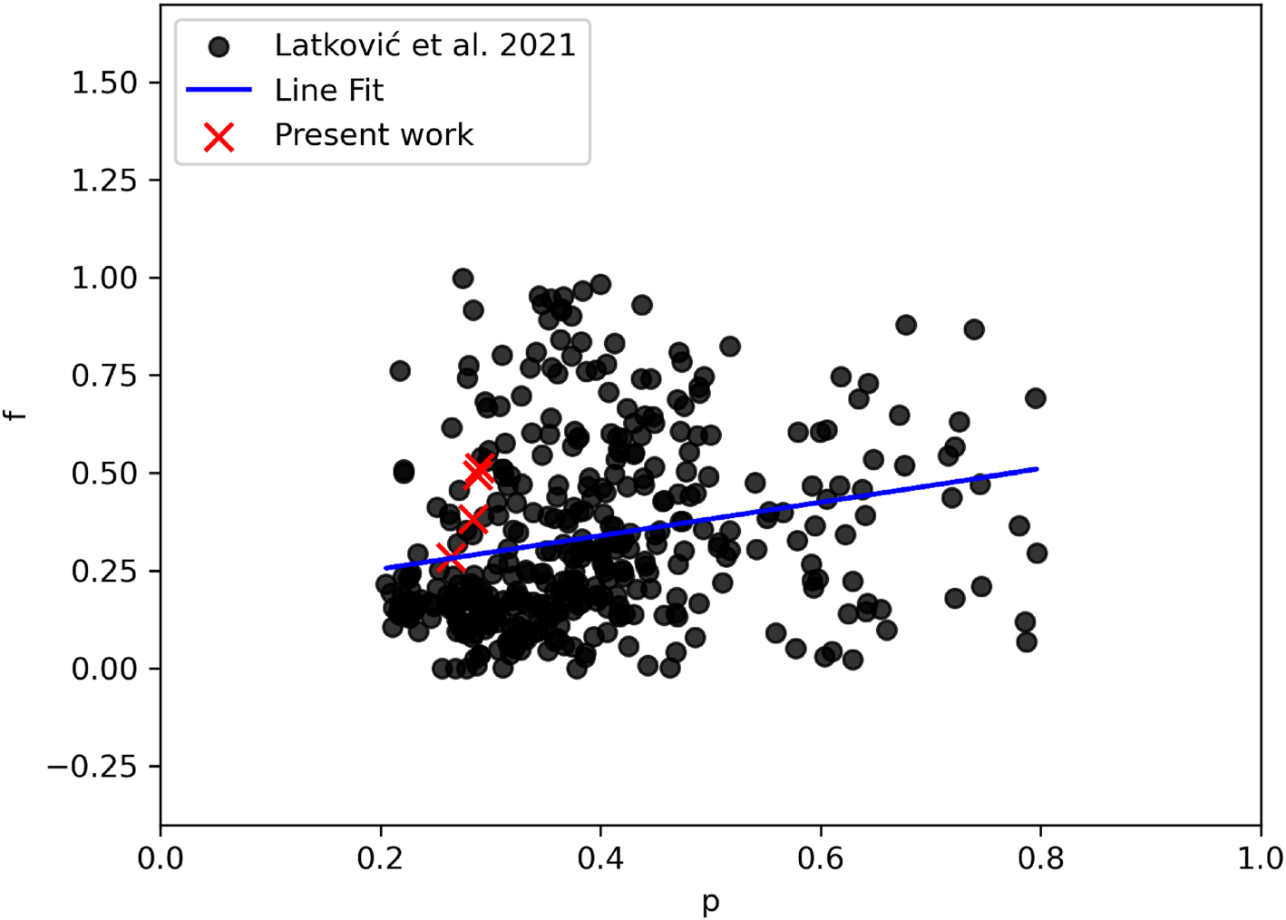
The relationship between periods (P) and fill-out ratios (*f*), with a linear regression line fit (blue line) for data points of W UMa systems with periods less than 0.8^d^. The black circles are the data collected from^48^. The red X signs are for the present work.

We determined the fill-out factor (*f*) for our systems using the method described by^49^, which is given by the equation *f* = $(\Omega _{\text {inner}} - \Omega ) / (\Omega _{\text {inner}} - \Omega _{\text {outer}})$. In this equation, $\Omega _{\text {outer}}$ and $\Omega _{\text {inner}}$ denote the outer and inner critical equipotential surfaces, respectively, while $\Omega$ represents the equipotential surface of the star. Our calculations yielded fill-out factor values of 49% ($\pm 2$), 38% ($\pm 1$), 28% ($\pm 2$), and 51% ($\pm 2$) for the four systems under study. These results suggest a moderate degree of contact configuration for these systems. For a visual representation of the system’s Roche geometry, refer to Fig. (4), which was generated using Binary Maker (BM3)^50^. Notably, we observed a proportional relationship between the fill-out ratio and the orbital period; systems with shorter periods exhibited lower fill-out ratios, and vice versa. Evidence of such correlation is observed when plotting the well-studied W UMa systems reported in^48^ (see Fig.5). This result may lend support to the explanation of a period cut-off observed in this class of eclipsing binaries, as reported by^21^.

In order to trace the evolutionary status for the presented systems, the absolute parameters for each system are calculated.

### M-L relation for EW systems

Several attempts have been made to get the mass luminosity relation of eclipsing binaries. These determinations from the early twentieth century see^51^ for a historical review.^52^ constructed a relation for EW type in particular.^51^ used a sample of 268 detached and double lined binary stars with accurate $\mathrm {T_{eff}}$ and luminosity measurements to improve the M-L relation. They divided the masses into four groups of low mass, intermediate mass, high mass and very high mass stars and each group has its mass-luminosity relation. Recently,^48^ made a statistical study on $\approx 700$ W UMa eclipsing binaries with published stellar parameters and concluded two equations for the mass luminosity relation for both the primary and secondary from linear fits (see their equations 9 and 10).

**Figure 6:**
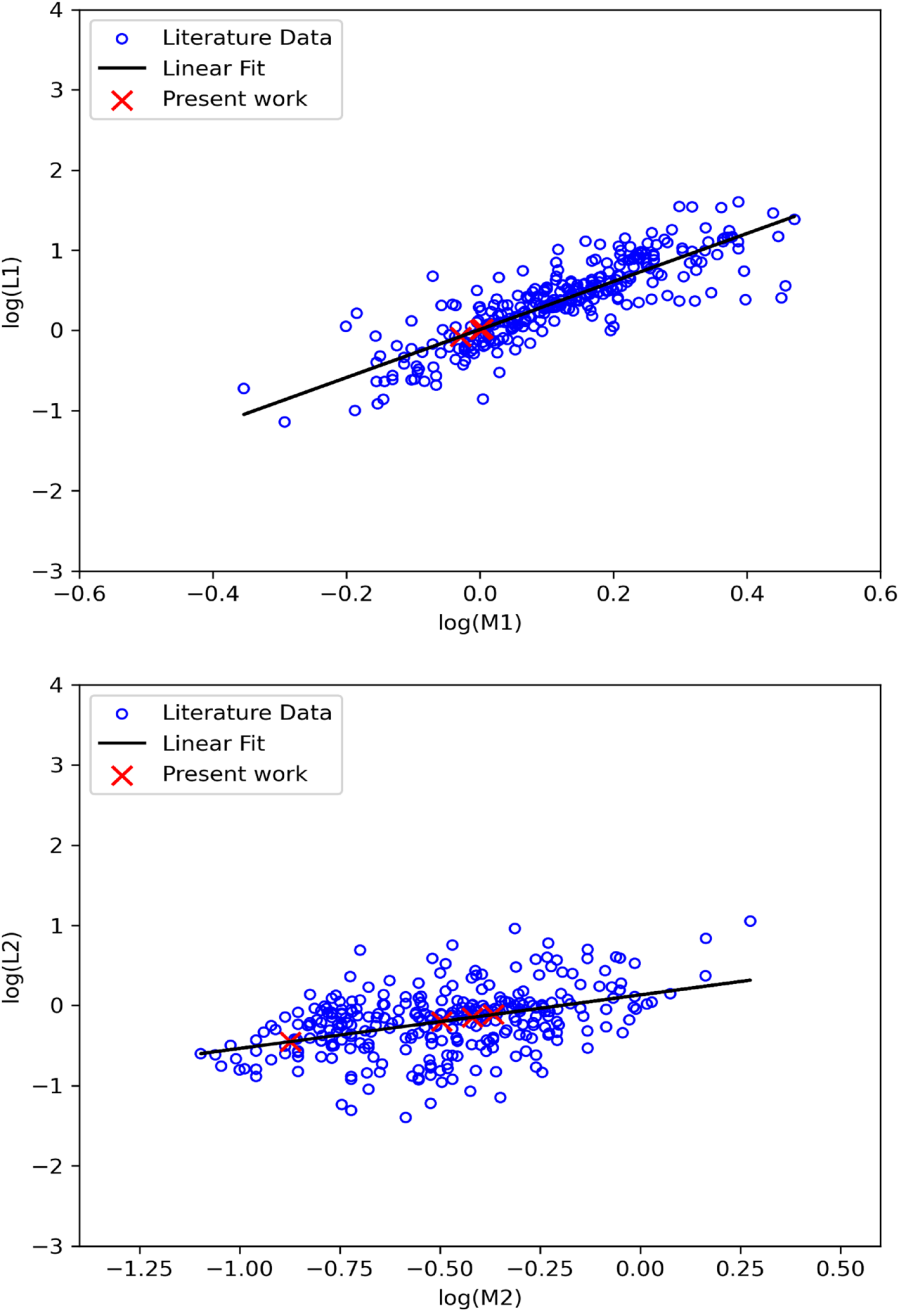
Mass-Luminosity Relation (M-L) for EW systems. The upper panel represents M-L for the primary components, while the lower panel is for the secondary ones. The blue circles are the data collected from^48^ and^53^, respectively. The red X signs are for the present work, while the solid black line marks our linear fit. The linear fit’s error is 0.01 and 0.02 for the upper and lower panel, respectively.

In the present work, we propose an update for these M-L relations for the EW UMa systems. Our sample is collected from previously published absolute parameters including^48^ and^53^. The selected sample is characterized by being spectroscopically observed or systems in total eclipse. This will help reduce the uncertainty in the estimation of the mass ratio and consequently the rest of the absolute parameters. Table 5 lists the first five rows of the data used in the present work, while the full version of the table can be found at 10.5281/zenodo.10209449.

Our linear fits resulted in the following relations,

5 $$\begin{aligned} \log L_1 & = (2.99 \pm 0.08) \log M_1 + (0.01 \pm 0.01) \end{aligned}$$

6 $$\begin{aligned} \log L_2 & = (0.67 \pm 0.04) \log M_2 + (0.13 \pm 0.02) \end{aligned}$$

Figure 6 reveals that the majority of absolute mass and luminosity values for W UMa components are concentrated within the range of 0.1 to 1.5 $M_{sun}$ for the secondary component, whereas for the primary components, they are found to be within the range of 0.6 to 2 $M_{sun}$. Compared to^51^, our results occupied the low and intermediate M-L domains.

**Table 5:** Absolute masses and luminosities of primary and secondary components of W UMa systems for illustrating the M-L relation in the current work.

No.	Name	M1	L1	M2	L2	Notes
1	1SWASP J034501.24+493659.9	0.654	1.632	0.275	0.775	^48^
2	1SWASP J044132.96+440613.7	0.703	0.121	0.448	0.071	^48^
3	1SWASP J052926.88+461147.5	0.804	0.358	0.331	0.163	^48^
4	1SWASP J064501.21+342154.9	0.7	0.23	0.3	0.135	^48^
5	1SWASP J074658.62+224448.5	0.79	0.241	0.28	0.118	^48^

### Absolute parameters and evolutionary status

To determine the absolute parameters of our systems, four empirically established mass-period relationships (M-PR) were employed to assess the mass of the primary star in W UMa binary systems. The initial M-PR was introduced by^54^, while subsequent relationships were developed by^55^ and^56^, and more recently by^48^. The average mass obtained from the four relations is designated as the mass ($M_1$) of the primary component, subsequently, we derived the mass of the secondary star based on the mass ratio obtained through light curve modeling. The semi-major axis ($a$) in solar radii ($R_{sun}$) was determined using Newton’s formulation of Kepler’s third law, where $(M_1 + M_2) = 0.0134 \cdot a^3/P^2$. With the effective radius ($r_i$) obtained from our light curve analysis and the estimated semi-major axis, we calculated the radii of both the primary and secondary stars in solar units as $R = r_i \cdot a$. To estimate the luminosities of the primary ($L_1$) and secondary ($L_2$) stars in solar units, we employed the Mass-Luminosity (M-L) relation presented in this study. Additionally, we computed the surface gravity ($\log (g)$) of the components using Eqs. (4) and (5) introduced by^9^. The parameter uncertainties were determined by considering the error bars associated with the relevant parameters.

**Figure 7:**
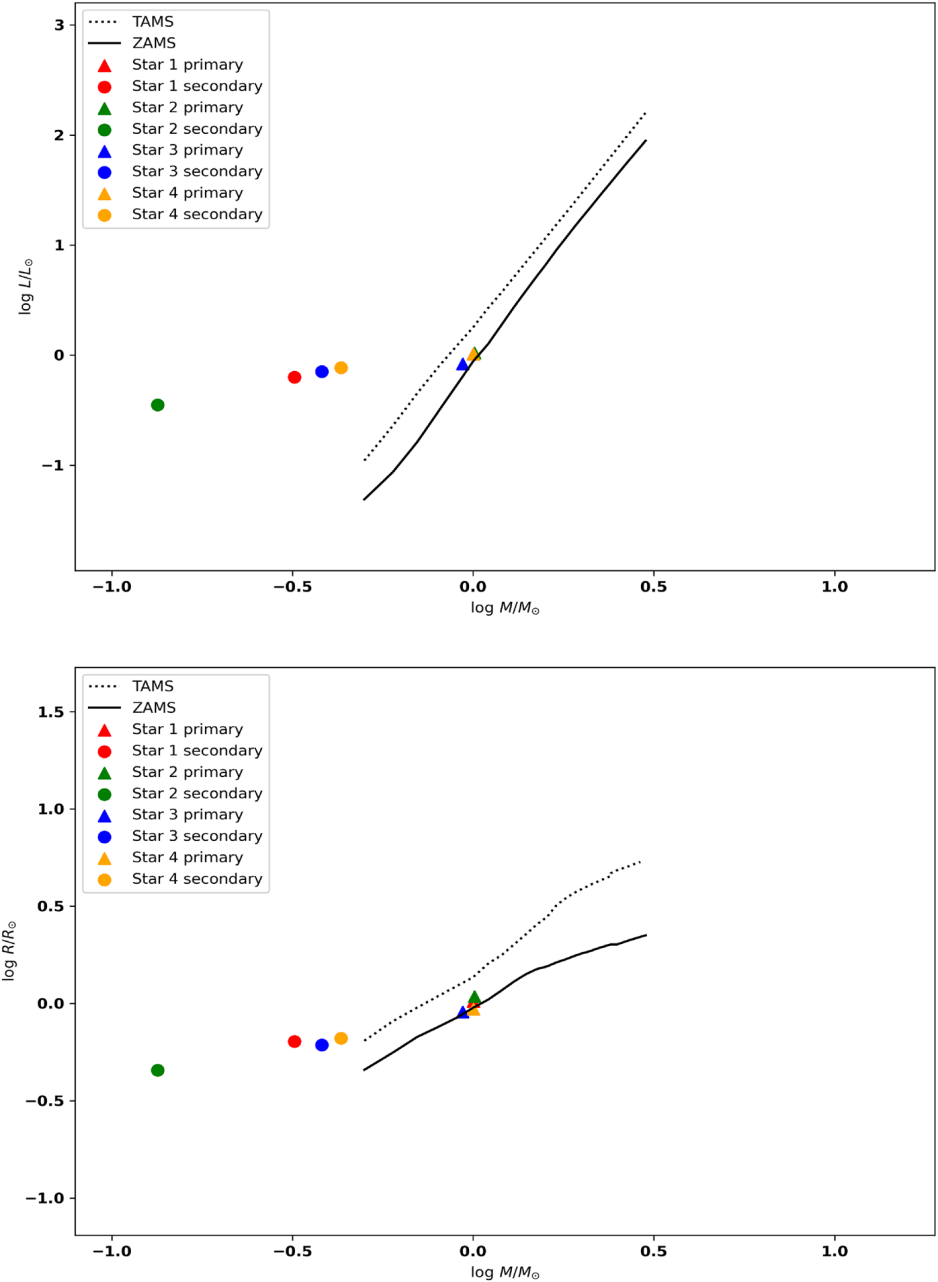
Mass-Luminosity (upper) and Mass-Radius (lower) evolutionary tracks for the four systems. The dashed lines represent the Terminal Age Main Sequence (TAMS), while the solid lines correspond to the Zero Age Main Sequence (ZAMS). These tracks were extracted from^57^.

**Figure 8:**
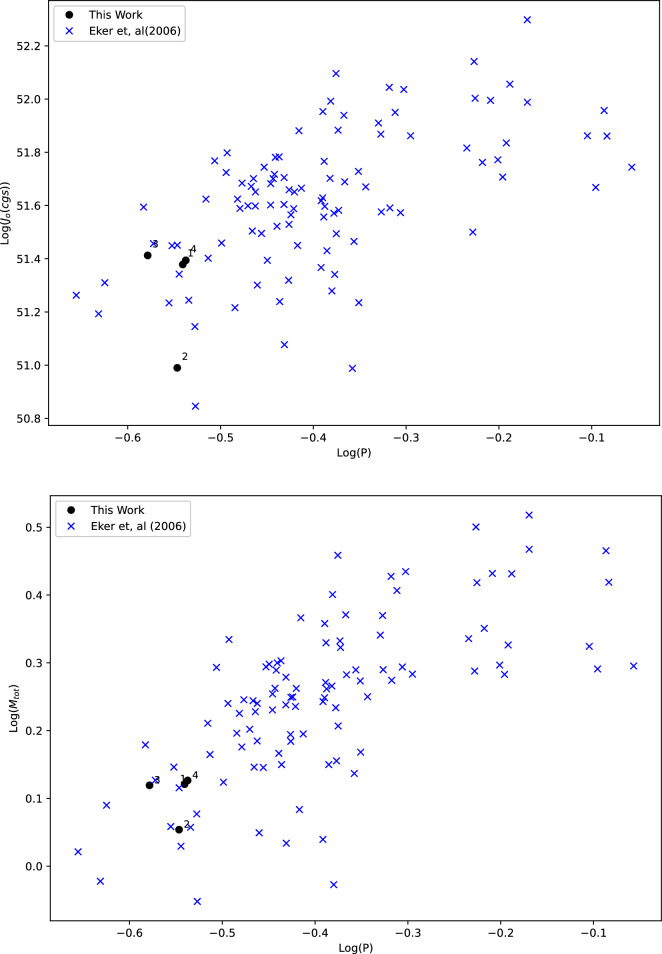
Period Angular momentum (upper) and Period Total mass (lower) plots for the four systems in comparison with^58^.

Utilizing the parameters listed in Table 6, we represented the evolutionary status of our systems on the Mass-Luminosity (M-L) and Mass-Radius (M-R) diagrams for the Zero Age Main Sequence (ZAMS) and Terminal Age Main Sequence Stars (TAMS). These diagrams were constructed based on the evolutionary tracks provided by^57^ with a metallicity (Z) of 0.014. The upper panel of Fig. 7 illustrates the positions of the four systems on the M-R track, while the lower panel of Fig. 7 displays the components on the M-L track. These figures clearly indicate that the primary stars in our systems primarily reside on the main sequence, indicating their relatively less evolved nature. Conversely, the secondary components of the four systems appear above the main sequence, signifying their evolved nature. Notably, the secondary component of S4 exhibits a relatively less evolved behavior compared to its counterparts, while S2 appears to be the most evolved component among them. Furthermore, we examined the dynamical evolution of the systems by studying the orbital angular momentum and total mass of the binary in relation to the orbital period, as investigated by^58^. We employed their Eq. (1) to calculate the orbital angular momentum for our systems. Additionally, we utilized Table 2 to construct the log(J_o_)-log(P) and log(M)-log(P) diagrams, as shown in Fig. 8. Our findings indicate that the four systems are positioned towards the lower left corners in both diagrams, as expected for W UMa stars.

**Table 6:** Absolute (global) parameters for each system.

Star ID	D (pc)	$L_1(L_{\odot })$	$L_2(L_{\odot })$	$R_1(R_{\odot })$	$R_2(R_{\odot })$	$M_1(M_{\odot })$	$M_2(M_{\odot })$	a$(R_{\odot })$	Log($g_1$)	Log($g_2$)	$J_o$
S1	517.32	1.026 (± 0.08)	0.632 (± 0.04)	1.026 (± 0.05)	0.638 (± 0.02)	1.001 (± 0.101)	0.320 (± 0.02)	2.014	4.50	4.380	1.81(± 0.06)
S2	1145.5	1.051 (± 0.06)	0.353 (± 0.046)	1.086 (± 0.042)	0.454 (±0.041)	1.009 (± 0.1)	0.134 (± 0.01)	1.902	4.506	4.385	0.80(± 0.03)
S3	455.74	0.837 (±0.04)	0.710(± 0.02)	0.906 (± 0.03)	0.612 (± 0.01)	0.935 (± 0.09)	0.381 (± 0.03)	1.898	4.534	4.412	0.3.24(± 0.09)
S4	607.07	1.025 (± 0.04)	0.770 (± 0.03)	0.935 (±0.03)	0.662 (± 0.01)	1.001 (± 0.10)	0.431 (± 0.04)	1.911	4.534	4.377	4.411 (± 0.1)

## Conclusion

In this study, we conducted a comprehensive analysis of four short-period eclipsing W UMa systems based on ZTF observations. By deriving absolute parameters such as luminosity, radius, mass, and surface gravity, we gained valuable insights into the physical properties and evolutionary status of these systems. The positioning of the systems on Mass-Luminosity and Mass-Radius diagrams indicated that the primary stars mainly reside on the main sequence, while the secondary components exhibit signs of evolution. Furthermore, the confirmation of the systems’ classification as W UMa binaries through dynamical evolution analysis strengthens our understanding of these systems. The detection of the O’Connell effect in one system (S4) allowed us to estimate spot parameters, revealing a hot spot on the surface of the secondary component, which could expand by mass transfer from the primary component to the secondary one. we also found that, the primary surface temperature as well as mass is higher than the secondary one, indicating A—sub type of W UMa systems. We updated the Mass-Luminosity relations through collecting a well studied sample with spectroscopic and/or totally eclipsed stars. This study also highlighted a relationship between the fill-out factor and the period, however this needs further investigation as well as observations, particularly near the period cut-off (i.e. less than 0.3^d^). The evolutionary status analysis demonstrated a noticeable evolution in the secondary components compared to the primary components in each system. Finally, we recommend conducting spectroscopic follow-up observations of these systems for further investigations and deeper insights into their properties.

## Data Availability

The data that supports the findings of this study is available at https://www.ztf.caltech.edu/ztf-public-releases.html.
